# Developing Biomarkers for Methamphetamine Addiction

**DOI:** 10.2174/157015911795017128

**Published:** 2011-03

**Authors:** John Mendelson, Matthew J Baggott, Keith Flower, Gantt Galloway

**Affiliations:** Addiction and Pharmacology Research Laboratory, California Pacific Medical Center Research Institute, St Luke’s Hospital 3555 Cesar Chavez San Francisco, CA 94110 USA

**Keywords:** Biomarker, methamphetamine, addiction.

## Abstract

There are an estimated 11.7 million methamphetamine (MA) abusers in the United States and epidemics of MA addiction are occurring worldwide. In our human laboratory and outpatient clinical trials we use innovative methods to quantify the severity of MA addiction and test biomarkers that may predict response to therapy or risk of relapse. One potential biomarker of addiction is the quantity of abused drug intake. Qualitative urinalysis is used in clinical trials and during treatment but provides only a binary outcome measure of abuse. Using non-pharmacologic doses of deuterium labeled l-MA we have developed a continuous quantitative measure to estimate the bioavailable amount of MA addicts ingest. Brain Derived Neurotrophic Factor is a neurotrophin that encourages growth and differentiation of new neurons and synapses. Low BDNF levels are seen in many addictive disorders and BDNF is elevated in recovering MA addicts, suggesting BDNF may be a marker of MA addiction. We are investigating the effects of controlled doses of MA on BDNF levels and gene regulation and measuring BDNF in our clinical trials. We believe both patients and clinical researches will benefit from the addition of new, objective and quantifiable outcome measures that reflect disease severity and recovery from addiction.

## INTRODUCTION

Addiction is a chronic disease characterized by harmful drug use and relapses after periods of sobriety. Many factors are thought to drive relapse, including stress, drug-associated cues and allostatic load. But, unfortunately, there are no reliable clinical signs or symptoms that predict relapse. This situation is not unique to addiction; many diseases lack clear cut, easily observable signs of disease progression or improvement. In the absence of pathomnemonic signs or symptoms of disease activity, analytical tools can be used to assess disease-associated biological parameters. These analytic measures are referred to as biomarkers.

Biomarker measurements have proven essential in clinical medicine. Many diseases are managed entirely thorough analysis of biomarkers. For example, CD4 cell counts and HIV viral loads (and not disease-defining criteria like opportunistic infections) are used to manage HIV. Biomarkers can help explain empirical results of clinical trials by relating clinical responses to the changes in molecular and cellular pathways. In doing so, biomarkers provide an avenue for researchers to gain mechanistic insight into differences in clinical response that may be influenced by uncontrolled variables (for example, drug metabolism) [1].

A biomarker is “a characteristic that is objectively measured and evaluated as an indicator of normal biological processes, pathogenic processes, or pharmacologic responses to a therapeutic intervention” [1]. We believe it will be impossible to develop addiction pharmacotherapies without discovery and validation of biomarkers.

In our laboratory, we are testing the utility of two biomarkers for predicting disease outcome and intensity. To help assess the response to therapeutic intervention we are using quantitative estimates of illicit MA intake. To assess normal and pathogenic biological processes, we are using an emerging biomarker of CNS function - Brain Derived Neurotrophic Factor (BDNF). Because our studies are ongoing results will not be presented here. In this paper we lay out the rationale for these biomarkers and discuss our strategy for developing addiction biomarkers.

Biomarkers are distinct from clinical and surrogate endpoints. Ideally a biomarker will track with clinical outcomes and accurately predict the future. However, many biomarkers that are useful in drug development never achieve the adequate predictive power of surrogate endpoints. Biomarkers may have the greatest value in early drug development studies. Biomarkers can be used to establish ‘proof of concept’, to separate patients with disease from those without disease, as a tool to stage disease, as an indicator of disease prognosis and to monitor the response of disease to therapy. Our biomarker development program is designed to establish proof of concept (BDNF), stage disease (quantitative estimates of MA intake), and monitor response to therapy (BDNF and quantitative estimates of MA intake).

## QUANTITATIVE ESTIMATES OF ILLICIT METHAMPHETAMINE EXPOSURE

Pharmacotherapy trials aim to discover medications that diminish illicit drug use. The ideal pharmacotherapy would produce abstinence without relapse (and be inexpensive and non-toxic). However, it is likely that multiple behavioral and medication interventions will be needed to complement one another to achieve a more moderate goal. Short of abstinence, a goal would be to decrease drug use, defined as a decline in both the frequency and amount of drug use. One objective measure of drug abuse is finding the drug (or, more commonly, a metabolite) in urine; this has become a standard tool. However, as currently used, this is non-quantitative and yields only a time-series of binary outcomes of “positive” or “negative.” 

The Consensus Statement on Evaluation of Outcome of Pharmacotherapy for Substance Abuse/Dependence, states that, although it can be very sensitive for detecting drug use in usually-abstinent individuals “[toxicology testing] is not sensitive in detecting either reductions in drug use or periods of up to 2-3 days abstinence in individuals who continue some use. The consequence has been that…extremely large reductions in use (e.g. perhaps up to 90% reductions in use) might be required before even a modest reduction in urinalysis-positive results could be expected.”

To address this problem, we have been developing a method to estimate the quantity of illicit drug exposure. Our method involves administration of small, pharmacologically inactive doses of deuterium-labeled l-methamphetamine (l-MA-d_3_). l-MA is the non-abused isomer of MA. Prior studies in our laboratory indicate that, at the doses we plan to give, l-MA is pharmacologically inactive [2-4].

In our outpatient trials, subjects will be given daily doses of a 5 mg of l-MA-d_3 _biomarker dose in addition to the experimental treatment medications. A urine sample is collected at each biweekly visit. The approximate time of ingestion of the last illicit dose is obtained using a timeline-follow-back procedure. Compliance with biomarker dosing is assessed by having subjects photograph pills just prior to ingestion. Pill photos are sent from cell phone to our server, giving a time and date for ingestion of the l-MA-d_3_ biomarker. Thus, data used for quantitatively estimating drug exposure are:

1. The dose and time of biomarker ingestion;
2. Urine concentrations of deuterium labeed l-MA-d_3_ and non-deuterated MA;
3. Time of last abused dose.

For renally-excreted drugs with first order elimination (like MA), urine concentration is directly related to plasma concentration. Although the concentration may vary with urine pH, age, creatinine clearance and other factors there will always be a constant relationship between plasma and urine concentrations. For our method, variability in elimination of MA does not affect estimation of the abused dose because deuterated MA (MA_deut_) and non-deuterium labeled MA (MA_non-deut_) have identical elimination [4-5]. Thus, the ratio of MA_deut_ to MA_non-deut _in the urine will be the same as the circulating ratios of MA. After distribution, circulatory concentrations of MA are directly related to CNS effect site concentrations. Thus, urine MA_deut_:MA_non-deut _is always a function of plasma MA_deut_:MA_non-deut_ and this ratio is directly related to concentrations at CNS effect sites. Variability in absorption could also affect quantitative dose estimation. However, we have shown that l-MA is completely absorbed (manuscript under review) and thus, the relative ratio between the deuterated and non-deuterated moieties reflects the relative ratios of MA available to act at CNS effect sites.

Our method does not estimate the amount of illicit MA used. It estimates the bioavailable amount —the proportion that actually has toxic effects— which will be some fraction of the amount abused. Thus, the method is robust against differences in purity and route of administration of illicit MA, eliminating variability in subject estimates of the quantity of drug abused. And our method is robust against differences in elimination, controlling for intraindividual factors such as urine pH and hydration status and interindividual variations in metabolism, weight, body fat, gender, and age.

Because there is no need to adjust for elimination or absorption, it is relatively simple to estimate the illicit absorbed effect site dose. We measure urine concentrations of MA_deut_, ([MA_deut_]) and [MA_non-deut_] and compare the ratio of [MA_deut_]:[MA_non-deut_]. To arrive at the estimated dose we adjust for time of dosing using a T_1/2_ of 12 hours, deriving a semi-quantitative estimated abused dose. The estimate is semi-quantitative because: 1) we do not know the true l_z_ (the rate constant for T_1/2_) of MA for each individual subject and 2) we assume the entire estimated dose was taken at the time of last abuse. This assumption simplifies a complex process of abuse where variable doses are abused over a hectic time course. However, due to the long half-life of MA, assigning total intake to the last known abused dose is reasonable, at least for a first approximation. There will also be a small but constant error in our estimate due to carryover and slow accumulation to steady state of the l-MA-d_3_ with daily dosing. We are aware of this and will use data from ongoing validation study to adjust estimates.

There are several scenarios where estimates would be severely biased or not possible. First, if there is little or no l-MA-d_3_ in the urine, it is likely that the subject has not been taking either the therapeutic medication or the biomarker. No estimate will be made in this case. Second, if subjects abuse MA close to specimen collection (within 2 hours) the abused dose may not be completely distributed, introducing bias. We will also not estimate an abused dose in this case. Readers might suspect that binge abuse would be hard to estimate. We have an ongoing laboratory trial to assess the variability introduced by a pseudo-binge (five small, hourly MA doses). For estimation purposes, we assign the total dose based on the time of last reported MA abuse. Readers should also note that we expect substantial variation in urine MA concentrations – indeed, the degree of variability has made quantitative estimation of intake seem impossible. However, we do not compare absolute levels but the ratio of levels, avoiding confounds from prior studies. 

## BRAIN DERIVED NEUROTROPHIC FACTOR

Brain Derived Neurotrophic Factor (BDNF) is the most abundant member of the neurotrophin family of trophic factors. BDNF encourages growth and differentiation of new neurons and synapses. It exerts its effects by binding to tropomyosin receptor kinase B (TrkB) receptor [6]. Antidepressants, acting through BDNF and TrkB, increase hippocampal neurogenesis, leading to the hypothesis that cellular neuroplasticity in the adult brain is necessary for the behavioral effects of antidepressants [7]. Thus, BDNF has emerged as an alternative to the monoamine hypothesis of depression – and, by extension, to addiction [8]. Amazingly, brain-derived BDNF can be measured in human serum, suggesting that BDNF may be useful as an indicator of potential for neuroplasticity. BDNF levels are low in the blood of depressed patients [9], those with work stress [10], and the hippocampus of suicide victims [11]. Levels rise during treatment with antidepressant [9] or electroconvulsive therapy in treatment resistant depression [12]. Low BDNF is associated with verbal memory impairment [13] and with cognitive impairment and dementia in women [14], while higher BDNF in the elderly is correlated with better performance on the mini mental status examination [15].

BDNF levels have been measured in several groups of addicts with the results differing between drug classes and drug use status at the time of study. BDNF is low in current smokers [16] and rises with smoking cessation [16, 17]. Cocaine, heroin, and cannabis addicts had low BDNF [18] although see [19]). Low BDNF is reported in alcoholism [20] and eating disorders [21, 22]. MA addicts with at least 30 days of sobriety had increased plasma BDNF [23]. These studies show addictive drugs can increase or decrease BDNF. However, studying only untreated addicts introduces many variables obscuring the specific effects of addictive drugs on BDNF.

Several animal studies show that BDNF levels and gene expression are altered by exposure to amphetamines. In rats, BDNF mRNA rises in several regions of the frontal cortex after a single exposure to non-neurotoxic dose of MA or cocaine – and these increases are followed by parallel increases in dopamine D_3_ receptor mRNA expression in the nucleus accumbens [24]. In contrast, *chronic* administration of MA or amphetamine (AMP) *decreases* BDNF levels in the hypothalamus and occipital cortex [25]. Compared to adult rats, MA given between postnatal days 11-14 increased BDNF above those produced by stress [26] and produced spatial learning deficits [27]. In a rodent study on interactions of amphetamine and closed brain injury, amphetamine increased brain BDNF [28].

We are studying plasma BDNF as a biomarker in methamphetamine addiction. Because BDNF is expressed by lymphocytes, we can track time-dependent changes in BDNF expression by PCR analysis [29]. In methamphetamine addicts with at least 30 days of sobriety, serum BDNF levels were increased [23]. This suggests BDNF rises during recovery from methamphetamine addiction. Unfortunately, no data are available on BDNF levels in active methamphetamine addicts or following controlled methamphetamine exposure.

On our studies to assess the use of BDNF as a biomarker in MA addiction trials we are determining dose and time dependent changes in BDNF after controlled oral doses of MA and interactions between BDNF on craving, mood and neurocognitive function. Animal and clinical data suggest there will be differences between dependent and non-dependent users. Thus, in our laboratory experiments, we study two populations of MA users – relatively light non-dependent abusers and non-treatment seeking dependent abusers – to obtain a fuller understanding of the relationships between drug exposure, addiction, mood and BDNF. In our outpatient clinical trials we use BDNF levels and gene expression as outcome variables. This synergistic approach will yield a rapid understanding on the relationship (if any) between BDNF and MA addiction.

## CONCLUSIONS

In this paper we have outlined the development of biomarkers of the amount methamphetamine abuse and recovery from methamphetamine addiction. Clinical trialists will clearly benefit from the addition of new, objective and quantifiable outcome measures that reflect disease severity and recovery from addiction. Validated biomarkers will also improve clinical decision making and patient care. Basic scientists can help this process by searching for novel markers of disease activity followed by active collaboration with clinical researchers.
